# Exploring Seaweed and Glycine Betaine Biostimulants for Enhanced Phenolic Content, Antioxidant Properties, and Gene Expression of *Vitis vinifera* cv. “Touriga Franca” Berries

**DOI:** 10.3390/ijms25105335

**Published:** 2024-05-14

**Authors:** Eliana Monteiro, Gabriella De Lorenzis, Valentina Ricciardi, Miguel Baltazar, Sandra Pereira, Sofia Correia, Helena Ferreira, Fernando Alves, Isabel Cortez, Berta Gonçalves, Isaura Castro

**Affiliations:** 1 Centre for the Research and Technology of Agro-Environmental and Biological Sciences (CITAB), University of Trás-os-Montes e Alto Douro (UTAD), 5000-801 Vila Real, Portugal; elianaribeiromonteiro@hotmail.com (E.M.); migueladbaltazar@gmail.com (M.B.); sirp@utad.pt (S.P.); sofiammcorreia@gmail.com (S.C.); helenaf@utad.pt (H.F.); icortez@utad.pt (I.C.); bertag@utad.pt (B.G.); 2 Institute for Innovation, Capacity Building and Sustainability of Agri-food Production (Inov4Agro), University of Trás-os-Montes e Alto Douro (UTAD), 5000-801 Vila Real, Portugal; 3 Department of Agricultural and Environmental Sciences—Production, Landscape, Agroenergy, University of Milan (UNIMI), Via G. Celoria 2, 20133 Milan, Italy; gabriella.delorenzis@unimi.it (G.D.L.); valentina.ricciardi@unimi.it (V.R.); 4 Symington Family Estates, Vinhos SA, Travessa Barão de Forrester 86, 4431-901 Vila Nova de Gaia, Portugal; fernando.alves@symington.com; 5 Department of Agronomy, University of Trás-os-Montes e Alto Douro (UTAD), 5000-801 Vila Real, Portugal; 6 Department of Biology and Environment, University of Trás-os-Montes e Alto Douro (UTAD), 5000-801 Vila Real, Portugal; 7 Department of Genetics and Biotechnology, University of Trás-os-Montes e Alto Douro (UTAD), 5000-801 Vila Real, Portugal

**Keywords:** *A. nodosum*, anthocyanin biosynthesis, antioxidant activity, berry quality, glycine betaine, transporter genes

## Abstract

Climate change will pose a challenge for the winemaking sector worldwide, bringing progressively drier and warmer conditions and increasing the frequency and intensity of weather extremes. The short-term adaptation strategy of applying biostimulants through foliar application serves as a crucial measure in mitigating the detrimental effects of environmental stresses on grapevine yield and berry quality. The aim of this study was to evaluate the effect of foliar application of a seaweed-based biostimulant (*A*. *nodosum*—ANE) and glycine betaine (GB) on berry quality, phenolic compounds, and antioxidant activity and to elucidate their action on the secondary metabolism. A trial was installed in a commercial vineyard (cv. “Touriga Franca”) in the *Cima Corgo* (Upper Corgo) sub-region of the Douro Demarcated Region, Portugal. A total of four foliar sprayings were performed during the growing season: at flowering, pea size, bunch closer, and veraison. There was a positive effect of GB in the berry quality traits. Both ANE and GB increased the synthesis of anthocyanins and other phenolics in berries and influenced the expression of genes related to the synthesis and transport of anthocyanins (*CHS*, *F3H*, *UFGT*, and *GST*). So, they have the potential to act as elicitors of the secondary metabolism, leading to improved grape quality, and also to set the foundation for sustainable agricultural practices in the long run.

## 1. Introduction

Vitiviniculture is one of the most important socioeconomic sectors in Portugal. From a global perspective, in the year of 2022, Portugal was the 10th largest wine producer and the 8th largest wine exporter [1]. Portugal has a total of 14 wine regions (12 in continental Portugal and 2 in the Azores and Madeira archipelagos) [2]. The Douro Demarcated Region (DDR), located in the northeast of Portugal, was recognized as the first demarcated wine region in the world. The DDR is the largest and the most heterogeneous mountainous wine region in the world, with a peculiar terroir, presenting Mediterranean-like climatic conditions with warm dry summers and mild wet autumns [3]. Portugal has many native cultivars, with “Touriga Franca” being the most utilized in the DDR to produce top-quality Port and table wines due to its rich phenolic composition (anthocyanins and flavanols) [4,5]. Ongoing climate change will present a challenge for the winemaking sector worldwide, but particularly in the warmest and driest regions of Southern Europe. In Portuguese vineyards, climate change will inflict progressively drier and warmer conditions and increase the frequency and intensity of weather extremes [6]. The use of foliar protection formulations have been considered to be a short-term adaptation measure to cope with climate change while improving fruit quality and have been used in different species, such as apples (*Malus domestica*) [7,8], hazelnut (*Corylus avellana*) [9,10], peaches (*Prunus persica*) [11], sweet cherries (*Prunus avium*) [12,13], strawberries (*Fragaria ananassa*) [14], and tomatoes (*Solanum lycopersicum*) [15]. Sprayed *Ascophyllum*-*nodosum*-based seaweed extracts are increasingly becoming one of the most-used biostimulants in agriculture, as well as the most studied. Improvements in the quality of berries through the regulation of the physiological, biochemical, and molecular processes in the grapevine have been reported [16,17,18,19,20,21]. Glycine betaine is naturally synthesized, non-toxic, and inexpensive, and it is considered one of the most-attractive biostimulants for plant stress protection since it can act as an osmoprotectant and can protect the photosynthetic machinery (photosystem II) and thylakoid membranes, alleviating cellular oxidative damage and stabilizing protein structures in several plants [22,23,24].

It is known that several biostimulants contain bioactive molecules called elicitors [21], which are all the signal molecules that are perceived and induce a defensive reaction in the plant, improving its ability to face adverse environmental conditions, acting on the primary or secondary metabolism [25,26]. In this way, to understand how *A*. *nodosum* and glycine betaine improve the quality of berries as a whole and, consequently, of wine, it is important to study the chemical and physical parameters of the berry. Bioactive compounds and antioxidant activities are relevant features of berry quality. Moreover, understanding the effect of biostimulants spraying on the biosynthesis of secondary metabolites, particularly anthocyanins, is extremely important in a red cultivar such as “Touriga Franca”. Nevertheless, the mechanisms of action and the effects of biostimulation on the secondary metabolism are not totally clear [27].

The aim of this study was to evaluate the effect of foliar application of a seaweed-based biostimulant (*A*. *nodosum*) and glycine betaine on berry quality, phenolic compounds, and antioxidant activity and to elucidate their action on berries’ secondary metabolism through the comparative transcriptomic analysis of key genes related to the biosynthesis and transport of anthocyanins [5,28,29,30,31,32].

## 2. Results

### 2.1. Berry Quality

**Figure 1:**
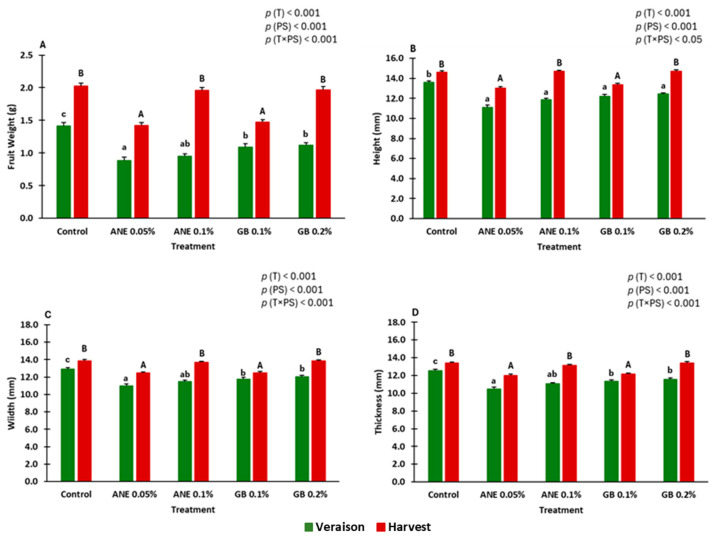
Biometric parameters: weight (**A**), height (**B**), width (**C**), and thickness (**D**) of berries of cv. “Touriga Franca” under different treatments at veraison and harvest. Values are means + SE; different letters mean significant differences (*p* < 0.05, Tukey’s test) between treatments within each phenological stage (lowercase—veraison; uppercase—harvest). ANE—seaweed extract; GB—glycine betaine; T—treatment; PS—phenological stage.

All the biometric parameters analyzed (weight, height, width, and thickness) were influenced by the foliar treatment, the phenological stage, and the interaction between treatment and phenological stage (0.05 < *p* < 0.001) (Figure 1A–D). The values of biometric parameters tended to be lower in the treated grapevines than in the control (C). At veraison, among the treated plants, those sprayed with glycine betaine (GB 0.1% and 0.2%) exhibited statistically significant and higher fruit weight, width, and thickness values compared to those treated with ANE 0.05% (*A*. *nodosum* extract). At harvest, significantly higher values for all the biometric parameters were also detected in grapevines treated with GB 0.2%, together with ANE 0.1% and C, compared to ANE 0.05% and GB 0.1%.

**Figure 2:**
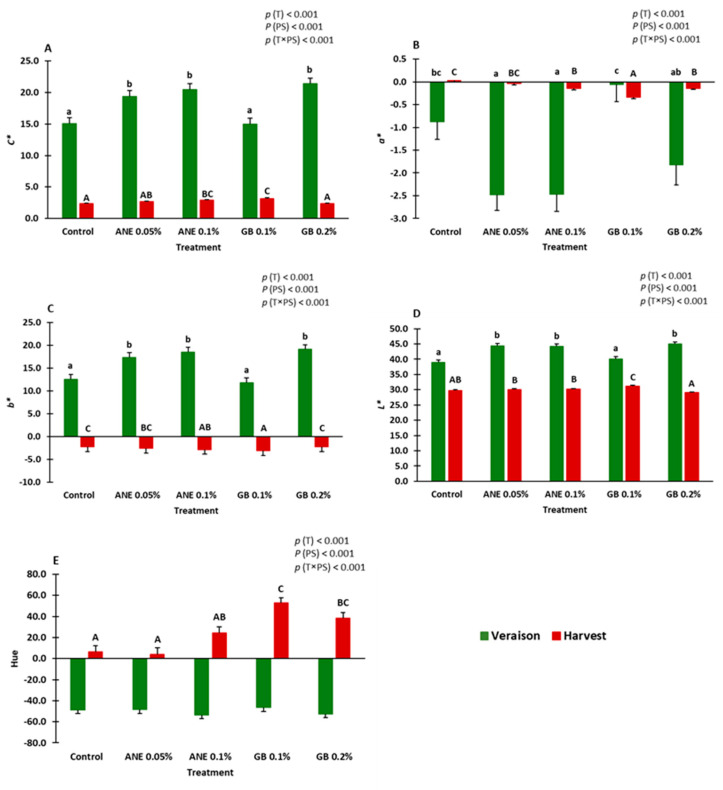
Chroma (C*) (**A**), coordinates a* (**B**), b* (**C**), L* (**D**), and hue (**E**) of berries of cv. “Touriga Franca” under different treatments at veraison and harvest. Values are means + SE; different letters mean significant differences (*p* < 0.05, Tukey’s test) between treatments within each phenological stage (lowercase—veraison; uppercase—harvest). ANE—seaweed extract; GB—glycine betaine; T—treatment; PS—phenological stage.

Chroma (C*), coordinates, and hue were influenced by treatment (*p* < 0.001), phenological stage (*p* < 0.001), and the interaction between treatment and phenological stage (*p* < 0.001) (Figure 2). At veraison, the berries treatment with ANE (0.05% and 0.1%) and GB 0.2% revealed a significantly higher C* compared to control and GB 0.1%. In the case of coordinate a* (indicating red (+a) to green (−a)), it was possible, as expected, to verify lower values at veraison than at the harvest. In the case of coordinates and b* (indicating yellow (+b) to blue (−b)), the opposite was verified. In the case of hue, high values at harvest in comparation to veraison were verified.

**Figure 3:**
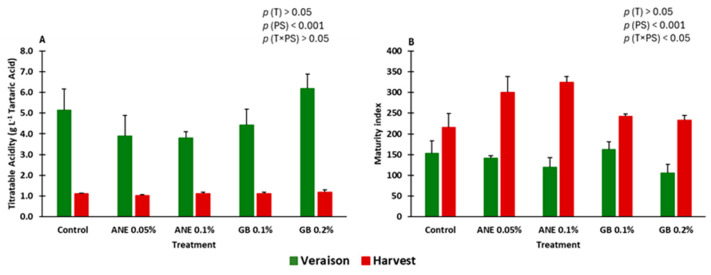
Titratable acidity (TA) (**A**) and maturity index (MI) (°Brix*pH^2^) (**B**) of berries of cv. “Touriga Franca” under different treatments at veraison and harvest. Values are means + SE; no letters indicate non-significant differences (*p* < 0.05, Tukey’s test) between treatments within each phenological stage. ANE—seaweed extract; GB—glycine betaine; T—treatment; PS—phenological stage.

Titratable acidity (TA) was only influenced by the phenological stage (*p* < 0.001) (Figure 3A).

In this study, MI (maturity index) was affected by the phenological stage (*p* < 0.001) and by the interaction between treatment and phenological stage (*p* < 0.05) (Figure 3B).

No statistically significant differences were found, neither at veraison or at harvest, between the treatments for TA and MI.

### 2.2. Phenolic Compounds

**Figure 4:**
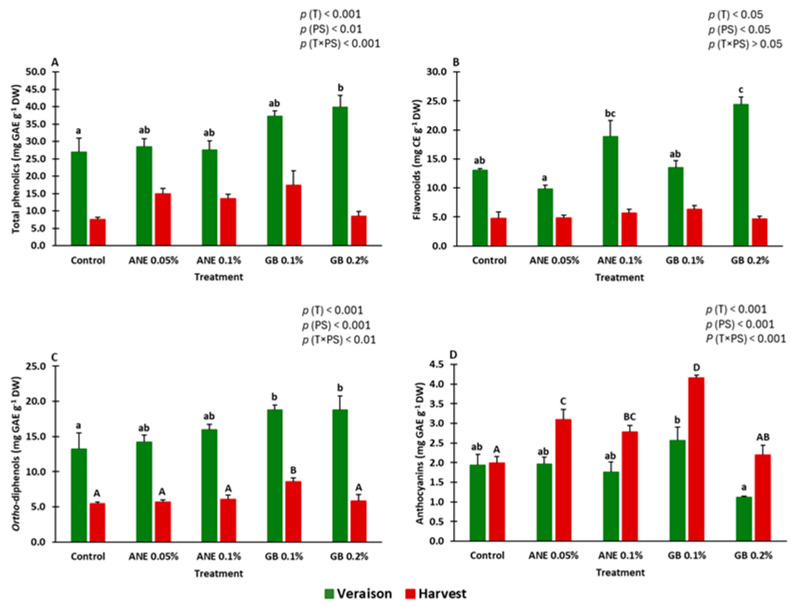
Variation in the content of phenolics compounds: total phenolics (**A**), flavonoids (**B**), *ortho*-diphenols (**C**), and total anthocyanins (**D**), in berries of cv. “Touriga Franca” under different treatments at veraison and harvest. Values are means + SE; different letters mean significant differences (*p* < 0.05, Tukey’s test) between treatments within each phenological stage (lowercase—veraison; uppercase—harvest); no letters mean no significant differences. ANE—seaweed extract; GB—glycine betaine; T—treatment; PS—phenological stage.

Overall, the treatments with GB enhanced the analyzed compounds (total phenolics, flavonoids, *ortho*-diphenols, and total anthocyanins) compared to the control (Figure 4) (see Supplementary Material, Table S1).

Total phenolics, flavonoids, *ortho*-diphenols, and total anthocyanins contents (Figure 4) were influenced by treatment (*p* < 0.001) and phenological stage (*p* < 0.01). Additionally, total phenolics, *ortho*-diphenols, and total anthocyanins contents were affected by the interaction between treatment and phenological stage (*p* < 0.001).

At veraison, GB 0.2% increased the concentrations of total phenolics, flavonoids, and *ortho*-diphenols by approximately 32%, 47%, and 29%, respectively, compared to the control (Figure 4A–C). At harvest, significant differences were observed in comparison to the control for *ortho*-diphenols and anthocyanin content, even in plants treated with GB (0.1%) (values 37% and 52% higher, respectively).

### 2.3. Antioxidant Potential

**Figure 5:**
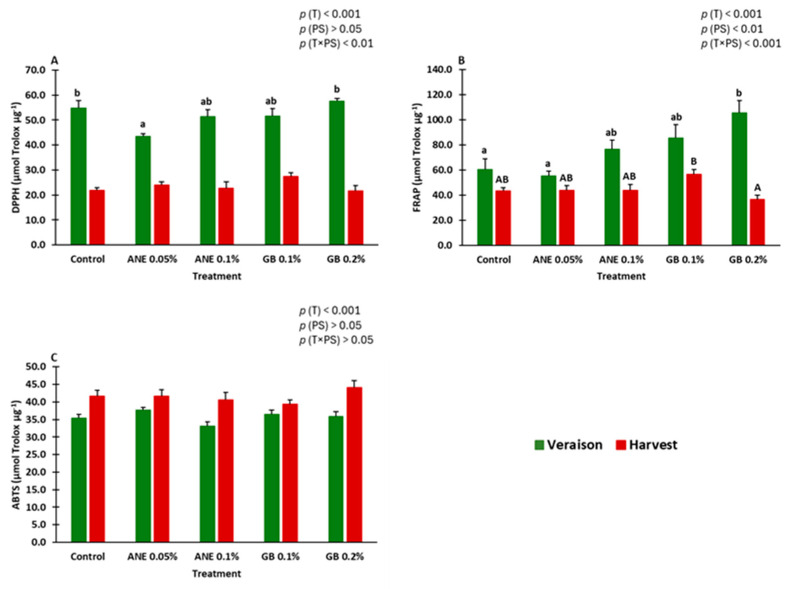
Antioxidant activity (AA): DPPH radical-scavenging activity (**A**), FRAP assay (**B**), and ABTS^•+^ radical-scavenging activity (**C**) in berries of cv. “Touriga Franca” under different treatments at veraison and harvest. Values are means + SE; different letters mean significant differences (*p* < 0.05, Tukey’s test) between treatments within each phenological stage (lowercase—veraison; uppercase—harvest) no letters mean no significant differences. ANE—seaweed extract; GB—glycine betaine; T—treatment; PS—phenological stage.

To assess the impact of foliar treatments on the antioxidant activity (AA) of berries, three different methods were used: DPPH, FRAP, and ABTS^•+^ (Figure 5) (see Supplementary Material, Table S2).

All the antioxidant activity results were affected by the treatment (*p* < 0.001) (Figure 5). Furthermore, the FRAP results were also influenced by the phenological stage (*p* < 0.01) (Figure 5B), and DPPH and FRAP by the interaction treatment x phenological stage (Figure 5A,C, respectively).

At veraison, the FRAP results indicated that GB 0.2% exhibited the highest increase in AA, approximately 43% higher than the control. For the remaining conditions, there were no significant differences among the various treatments in terms of antioxidant AA results.

### 2.4. Gene Expression

**Figure 6:**
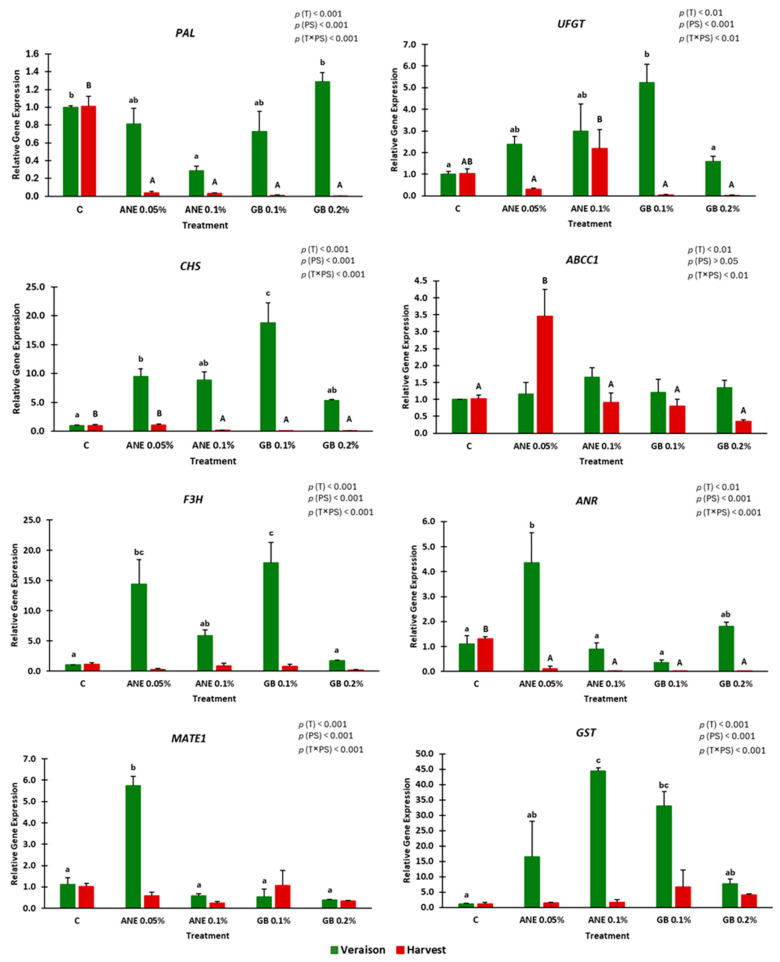
Relative gene expression (*PAL*, *CHS*, *F3H*, *MATE1*, *UFGT*, *ABCC1*, *ANR*, and *GST*) in berries of cv. “Touriga Franca” under different treatments at veraison and harvest. Values are means + SE; different letters mean significant differences (*p* < 0.05, Tukey’s test) between treatments within each phenological stage (lowercase—veraison; uppercase—harvest); no letters mean no significant differences. ANE—seaweed extract; GB—glycine betaine; T—treatment; PS—phenological stage.

To investigate the impact of the tested biostimulants on the secondary metabolism level, known to enhance the content of phenolic compounds, we analyzed the expression of several target genes encoding key enzymes involved in flavonoid biosynthesis and transport. These genes include *PAL*, *CHS*, *F3H*, *ANR*, *UFGT*, *ABCC1*, *MATE1*, and *GST* in berries at veraison and harvest (Figure 6) (see Supplementary Material, Table S3). The relative gene expression was influenced by the treatment (0.01 < *p* < 0.001), by the phenological stage (*p* < 0.001) (except for *ABCC1*), and by the interaction between treatment and phenological stage (0.01 < *p* < 0.001).

*Ascophyllum*-*nodosum*-based extract at a concentration of 0.1% (ANE 0.1%) led to significant differences in the relative gene expression of *GST* compared to the control at veraison. In the case of ANE 0.05%, it affected the expression of *CHS*, *F3H*, *ANR*, and *MATE1*. On the other hand, GB 0.1% promoted the up-regulation of *CHS*, *F3H*, *UFGT*, and *GST*. At harvest, *ABCC1* was up-regulated in berries treated with ANE 0.05%, and UFGT was up-regulated by ANE 0.1%.

## 3. Discussion

Several studies have shown that the foliar application of ANE and GB can influence the physical and chemical characteristics of fruits [12,14,16,17,18,19,33,34,35]. In this study, berries of grapevines sprayed with GB 0.2% were bigger and heavier in relation to the ANE 0.05% berries. Studies using GB also report this trend in grapevine [36] and other species, namely, strawberry [14], sunflower achenes [33], sweet cherry [12,13], and olive [35].

Color is an important attribute in the cultivar under study, as this cultivar is widely used in the production of Port and high-quality table wines. During this study, it was verified that the parameter C* was lower in berries treated with GB 0.1% (at veraison) and GB 0.2% (at harvest). At veraison, GB0.1% also showed the higher value for coordinate a*, and at harvest, this treatment showed high values for hue. Considering that C* refers to color saturation, higher C* values are indicative of non-colored berries, whereas lower C* values are linked to colored ones [14,37]. In the case of coordinate a*, this indicates red (+a) to green (−a) colors. In the case of hue, lower values were associated with non-colored berries, and the higher values were correlated with colored berries. These results suggest that GB can enhance the color quality of grapes. Similar results have been observed not only in grapevines, but also in other fruit species, including sweet cherry [12,13] and strawberry [14].

The acidity of berries can be influenced by water availability and high temperatures during berry ripening, which can have implications for wine quality [6,38]. Regarding foliar spraying’s effect on berries acidity, when comparing the behavior of one single cultivar in the same terroir conditions, the phenological stage has a more pronounced effect over treatments. In our previous study with the same biostimulant treatments and cv. Touriga Franca, in the upper-Douro sub-region of DDR, we have verified a decrease in the TA from veraison to harvest over two growing seasons (2020 and 2021) [36]. In this study, TA was affected by the phenological stage (*p* < 0.001), and it decreased, as expected, from veraison to harvest (Figure 3A). No significant differences were found between the treatments at the statistical level. Also, Frioni et al. [8], applying ANE in the “Sangiovese” grapevine cultivar, observed no significant differences in TA between treated and untreated grapevines. At veraison, which marks the onset of ripening, grapes undergo physiological changes, including the accumulation of sugars and the degradation of organic acids. The decrease in TA occurs primarily due to the breakdown of malic and tartaric acids, the two main organic acids found in grapes. Malic acid, in particular, is metabolized into simpler compounds such as lactic acid and pyruvic acid, contributing to the overall reduction in acidity.

The decrease in acidity also influences the maturation index (MI) values as pH is one of the factors considered in the calculation of the index. It is expected that the MI values would increase from veraison to harvest due to the combination of rising sugar content and decreasing pH, reflecting the progression of grape ripeness. Therefore, the MI parameter is commonly employed to determine the optimal time for harvest. The treatments under study did not significantly affect the MI (Figure 3A), as we previously observed in a different vineyard of cv. Touriga Franca in 2020 and 2021 [36]. As expected, the MI was influenced by the phenological stage (*p* < 0.001). Weather conditions can influence the MI, as observed in grapevine by Rätsep et al. [39]. In Portugal, the year 2020 was notably hot and dry, resulting in an advancement of the veraison and harvest phenological stages. This advance was not only evident compared to 2019 but also in comparison to the six-year average from 2014 to 2019. Across the three sub-regions of the DDR, the advance for veraison varied from 6 to 8 days compared to the 6-year average, while for harvest, it ranged from 9 to 15 days [40].

It is well established that biostimulants can enhance the vigor, plant yield, fruit quality, antioxidant capacity of plant tissues, nutrient uptake, and distribution within the plant, and they can also bolster tolerance to biotic and abiotic stress [41,42]. Biostimulants contain elicitors which can induce the activation of enzymes involved in primary or secondary metabolism, leading to, for instance, an increase in the synthesis of phenolic compounds [26,43]. However, the limited information regarding the mode of action of the studied biostimulants, namely, ANE and GB, and the mechanisms of grapevine responses to their application calls for further research. In this study, the effects of both extracts on grapevine were evaluated under field conditions, and it was verified that they increased the synthesis of anthocyanins and other phenolics in berries (Figure 4).

During the study, it was verified that the concentration of phenolics (total phenols, flavonoids, and *ortho*-diphenols) (Figure 4) and the AA (DPPH and FRAP) were higher at the veraison stage compared to the harvest (Figure 5). The same trend was observed in the study of the relative gene expression of the genes *PAL*, *CHS*, *F3H*, *MATE1*, *UFGT*, *ABCC1*, *ANR*, and *GST* (Figure 6), which were generally down-regulated at harvest. Veraison represents a critical phenological stage in red grape cultivars, initiating the accumulation of phenolic compounds and anthocyanins responsible for color development [31]. Higher mean temperatures at veraison in July (28 °C) and lower at harvest in September (22 °C) may have contributed to these contents’ patterns. The bunch exposure to high temperatures and radiation may increase flavonoids synthesis in grapes due to increased activity of the *PAL* [44]. 

The studied genes encode enzymes involved in key metabolic steps of the secondary metabolism. *Phenylalanine ammonia lyase* (*PAL*) initiates the phenylpropanoid pathway [5,45]. In this study, we verified that *PAL* is up-regulated by control (Figure 6). In the case of treatments, it appears that the concentration of anthocyanins increases faster; therefore, the expression of this gene decreases at harvest. However, the accumulation of anthocyanins begins at the same time in treatments and C, and the accumulation is continuous until harvest, but based on the data obtained, treatments appear to be faster in their accumulation. Other important genes encode enzymes that serve as intermediates in the production of colorless anthocyanins are *CHS*, *F3H*, and *ANR*. They are responsible for the synthesis of proanthocyanidins, also referred to as condensed tannins [5,29,30,31,32]. At veraison, it was verified that the treatments ANE 0.05% and GB 0.1% exhibited a more-pronounced influence on these genes during veraison (Figure 6), with a corresponding increase in flavonoids, *ortho*-diphenols, and anthocyanins concentrations compared to the control (Figure 4). These findings suggest that the studied biostimulants induce the flavone synthesis at the molecular level in cv. “Touriga Franca”. Similar outcomes were observed in grapevines with kaolin application, which up-regulated the *CHS* gene and enhanced flavonoid and anthocyanin synthesis at maturity [5,29], as well as with chitosan, which up-regulated the *F3H* gene [31]. *UFGT* mediates the limiting step towards anthocyanin biosynthesis and is associated with anthocyanins accumulation [31]. GB 0.1% up-regulated *UFGT* during veraison (Figure 6), coinciding with an increase in anthocyanin content for this treatment (Figure 4D). Singh et al. [31], similarly, found an increase in anthocyanin content and up-regulation of *UFGT* in treated vines of the cv. “Tinto Cão” following foliar application of chitosan. Anthocyanins are stored in the vacuole and are transported by *anthocyanin transporter* (*ABCC1*), *tonoplast transporter* (*MATE1*), and *glutathione S-transferase* (*GST*) [5,31]. The gene *ABCC1* did not show significative differences (*p* > 0.05) between treatments at veraison (Figure 6), a finding consistent with the lack of significant changes observed for this gene in the grapevine cv. “Tinto Cão” following chitosan application [31]. At harvest, the treatment ANE 0.05% up-regulated their expression, being one of the treatments with more anthocyanin concentration. The up-regulation on the expression of the key transporter gene *GST* at veraison in treatments ANE 0.1% and GB 0.1% was accompanied by higher concentrations of flavonoids (ANE 0.1%) and *ortho*-diphenols (GB 0.1%), as well as significantly higher anthocyanins content (GB 0.1%) (Figure 4). Frioni et al. [17], similarly, observed an increase in total anthocyanins, phenolic concentration, and gene expression (*UFGT*, *LDOX*, *GST*, *F3’H*, *F3’5’H*, and *DFR*) through the foliar application of ANE.

**Figure 7:**
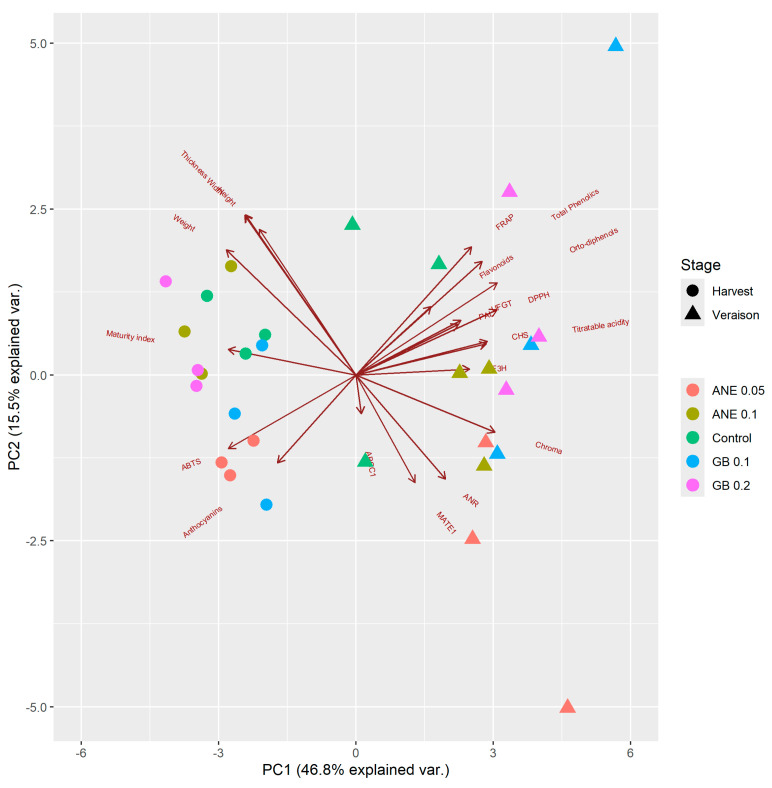
Principal component analysis (PCA) of different berry-related traits. ANE—seaweed extract; GB—glycine betaine.

For the PCA, the first two principal components (PCs) explained about 62% of total variance (Figure 7). It was possible to see the separation between the samples of veraison and harvest.

**Figure 8:**
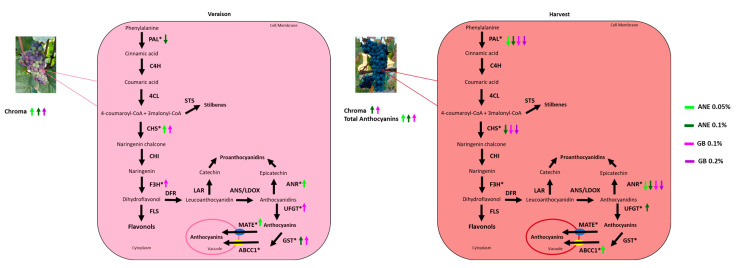
Phenylpropanoid and flavonoid pathways in grape berry cells. The molecular mechanisms studied in the present work are represented by genes marked with *, and the arrows highlight the up-regulation and down-regulation of transcripts. ANE—seaweed extract; GB—glycine betaine.

The use of biostimulants such as ANE and GB can serve as a short-term adaptation measure to cope with climate change, enhancing berry quality traits, phenolic compounds, and the up-regulation of associated genes (Figure 8), thus improving antioxidant activity, as demonstrated in this study. These results suggest a potential positive elicitation effect for both treatments under study.

It was possible to verify that the parameters flavonoids, total phenolics, *ortho*-diphenols, antioxidant activity (DPPH, FRAP, and ABTS^•+^), MI, biometric parameters (height, width, and thickness), and chroma were highly correlated at both phenological stages (Figure S1). The parameters TA, berry weight, and anthocyanins content were more correlated at harvest (Figure S1), when bigger berries were verified (Figure 1) with higher anthocyanins concentration (Figure 4D). *PAL* seems to be less correlated in general, with exception for GB 0.2% at veraison and control at harvest (Figure S1), which is in concordance with its expression, up-regulated in both treatments (Figure 6). The genes *CHS*, *F3H*, *UFGT*, and *GST* were down-regulated at harvest (Figure 6) and, at the same time, less correlated in this stage (Figure S1, see Supplementary Material). *GST* showed the same correlation pattern at veraison and harvest (Figure S1).

The general correlations (Figure S2, see Supplementary Material) analyzed between the berry-related traits in both phenological stages allowed us to verify that total phenolics were positively correlated with *ortho*-diphenols (0.88 ***), DPPH (0.78 ***), and FRAP (0.77 ***) (Figure 3, Figure 4 and Figure 5). The same trend was observed for *ortho*-diphenols: positively correlated with DPPH (0.88 ***), FRAP (0.88 ***), and TA (0.79 ***) (Figure 3, Figure 4 and Figure 5), and negatively correlated with ABTS^•+^ (−0.78 ***). DPPH showed a positive correlation with FRAP (0.80 ***) and TA (0.88 ***). Both FRAP and DPPH had a negative correlation with ABTS^•+^ since they presented opposite behavior (Figure 5). TA had a positive correlation with chroma (0.84 ***) as both have decreased from veraison to harvest (Figure 2 and Figure 3). The biometric parameters (weight, height, width, thickness) were strong and positively correlated between each other (0.82 *** to 1.00 ***). Chroma showed a negative correlation with biometric parameters as chroma decreased from veraison to harvest and biometric parameters increased (Figure 1 and Figure 2). The genes *CHS* and *F3H* showed a strong positive correlation (0.92 ***), as well as the genes *CHS* and *UFGT* (0.82 ***), having the same expression patterns (Figure 6 and Figure S1). In the case of the genes *ANR* and *MATE1*, they were also positively correlated (0.82 ***), with the same expression pattern, and with ANE 0.05% being the treatment with more influence on both genes at veraison. 

## 4. Materials and Methods

### 4.1. Plant Material and Sampling

**Figure 9:**
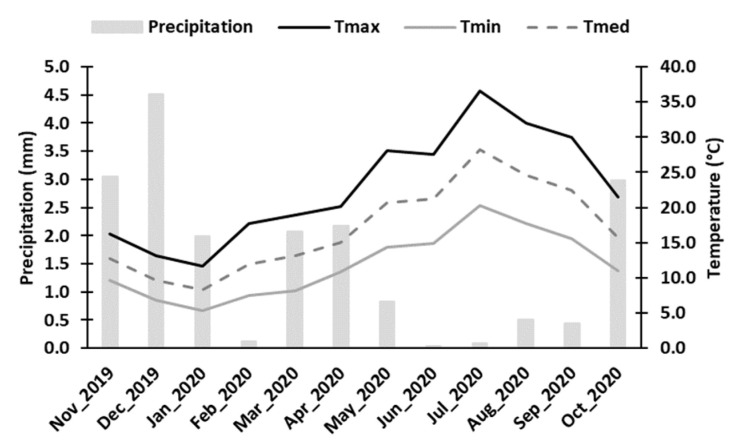
Monthly mean climatic conditions occurred during growing season in the vineyard Quinta do Bomfim in the *Cima Corgo* sub-region. Precipitation (mm); maximum temperature—Tmax (°C); minimum temperature—Tmin (°C); mean temperature—Tmed (°C).

Samples were obtained from *Vitis vinifera* cv. “Touriga Franca”, grafted on R110, in the growing season of 2020. The trial was installed in the *Cima Corgo* (Upper Corgo) sub-region of the Douro Demarcated Region, Pinhão, Portugal (41°11′30.7″ N 7°32′10.7″ W, 170 m above sea level). Row and vine spacing was 2.50 m and 0.80 m, respectively, and vines were trained via unilateral Royat cordon with vertical shoot positioning (VSP) in an east–southeast to west–northwest orientation. The vineyard was in rainfed conditions and grown using standard cultural practices of the region. Monthly temperature and precipitation values were recorded by a weather station located near the experimental site and are shown in Figure 9.

Three vineyard rows were sprayed with the following treatments: *A*. *nodosum* (in the form of the seaweed-based extract SPRINTEX NEW^®^ L, Biolchim, containing a high concentration of naphthaleneacetic acid, amino acids, and extract of *A*. *nodosum*) (ANE) at two concentrations (ANE 0.05% and ANE 0.1%); glycine betaine (Greenstim^®^, Massó Agro Department, containing (*w*/*w*) 12% of total N, 11.5% organic N, 56% organic C, and a relation C/N of 4.9, as a concentrate of glycine betaine extracted from sugar beet) (GB) at two concentrations (GB 0.1% and GB 0.2%); and control (C, water) (5 treatments × 10 plants × 3 replicates) (Table S4 see Supplementary Material). The concentrations used were determined through a combination of manufacturer recommendations and preliminary experimentation to prevent any adverse effects. All applications were mixed with a wetting agent (0.1%). SPRINTEX NEW^®^ L and Greenstim^®^ were commercialized according to the national legislation decree-law 103/2015 of June 15th. Currently, only Greenstim^®^ is part of the list of non-harmonized fertilizing materials authorized for organic viticulture, with registration valid until 2028, as requested by EU and national regulations (EU 2019/109 of June 5th and Ordinance 185/2022 of July 21st), respectively. Foliar applications were conducted during the morning, covering the whole canopy, with a total of four foliar sprayings: at flowering (BBCH 65), pea size (BBCH 75), bunch closer (BBCH 77), and veraison (BBCH 81) [46]. At veraison and harvest, berries were randomly sampled from the middle section of the clusters. Of these, 90 berries were selected and used for quality analysis (i.e., divided into three replications of 30 berries). For the analyzed global phenolic parameters, antioxidant activity determinations, and gene expression analysis, three replicates of berries per treatment were sampled and immediately frozen in liquid nitrogen until conservation at −80 °C and then lyophilized and converted to a fine dried powder (ground with liquid nitrogen) before the laboratorial analysis.

### 4.2. Quality Assessment of Fruit

To assess the impact of foliar treatments on the quality, 90 fresh berries were sampled at veraison and harvest stages. Biometric parameters (berry weight and dimensions), color, total soluble solids, pH, and titratable acidity were determined. For biometric parameters, fruit weight (g) was determined using an electronic balance, and the height, width, and thickness (mm) measured using a digital caliper (0.01 mm sensitivity).

Using a colorimeter (CR-300, Minolta, Osaka, Japan), the external fruit color was assessed at opposite sides of each fruit (a total of 90 berries per treatment). The colorimeter was calibrated using a standard white plate. The colorimetric coordinates L*, a*, and b* were used to calculate the chroma (C* = (a*^2^ + b*^2^)1/2) and the hue values (hue = Arc Tan (b/a)). The total soluble solids (TSS in °Brix) of berries’ juice were determined using a portable refractometer (PAL-1, ATAGO, Tokyo, Japan), the pH using a portable pH meter (Hanna instrument, USA), and the titratable acidity (gL^−1^ tartaric acid) via a manual glass burette using 0.1 M NaOH to an endpoint of pH 8.1 in 10 mL of juice diluted in 10 mL distilled water [47]. Finally, the maturity index (MI) was calculated according to Coombe et al. [48], using the following formula: MI = TSS*pH^2^.

### 4.3. Determination of Phenolic Compounds

To assess the impact of foliar treatments on the phenolics of berries, parameters such as total phenolics, flavonoids, *ortho*-diphenols, total anthocyanins, and antioxidant activity (ABTS^•+^, DPPH and FRAP) were determined according to the method of Singleton and Rossi [49] and Dewanto et al. [50].

The berries extracts were obtained according to Singleton and Rossi [49] and Dewanto et al. [50]. For this, 40 mg of dry material were mixed with 950 µL of 70% (*v*/*v*) methanol in a vortex; then, the mixture was submitted during 30 min to 70 °C and finally centrifuged at 13,000 rpm at 1 °C for 15 min. These extracts were stored at −20 °C and used for the determination of the total phenolics, flavonoids, *ortho*-diphenols, and in antioxidant activity (AA) assays.

#### 4.3.1. Total Phenolics

The total phenolics concentration was determined using the Folin–Ciocalteu colorimetric method at 765 nm according to Singleton and Rossi [49]. For this, 20 μL of extract was mixed with 100 μL of Folin–Ciocalteu reagent (1:10) and 80 μL of Na_2_CO_3_ (7.5%) in a 96-well microplate. The microplate was maintained in the dark for 30 min; then, the absorbance values were obtained at 765 nm. A gallic acid calibration curve was used, and the results were expressed as mg of gallic acid equivalents per g of dry weight (mg GAE g^−1^ of DW).

#### 4.3.2. Flavonoids

According to the colorimetric method of Dewanto et al. [50], flavonoids concentration was determined at Abs. 510 nm. A 96-well microplate was supplemented with 100 μL of ddH_2_O, 10 μL of NaNO_2_ (5%), and 25 μL of extract. The plate was placed in the dark at room temperature for 5 min; then, 15 µL of AlCl_3_ (10%) was added to each well, and the plate was placed in the dark again for 6 min. Finally, 50 μL of NaOH (1 M) and 50 μL of ddH_2_O were added and the absorbance was read. Using a calibration curve prepared with catechin, the results were expressed as mg of catechin equivalents per g of dry weight (mg CE g^−1^ of DW).

#### 4.3.3. Ortho-Diphenols

The *ortho*-diphenols were quantified according to Gouvinhas et al. [51] and Leal et al. [52] using a colorimetric method at Abs370 nm. For that, in a 96-well microplate, 160 μL of extract was mixed with 40 μL of sodium molybdate (5% *w*/*v*), and the plate was placed in the dark for 15 min. A calibration curve prepared with gallic acid was used, and the results were expressed as mg of gallic acid equivalents per g of dry weight (mg GAE g^−1^ of DW).

#### 4.3.4. Total Anthocyanins

The total monomeric anthocyanins (TMA) content was determined according to Lee et al. [53], Meng et al. [54], and Ali Shehat et al. [55]. For the extracts preparation, 5 mL of methanol acidified with 1% HCl was mixed in a vortex with 50 mg of berries. The mixture placed in the dark for 1 h at 4 °C, and after that, it was centrifuged at 4000 rpm for 15 min at 4 °C, and the supernatant was collected. In a microplate, a mixture of 50 μL of extract plus 250 μL of 0.025 M KCl (pH = 1.0) or 50 μL of extract plus 250 μL of 0.4 M sodium acetate buffer (pH = 4.5) was pipetted into two different wells. Finally, absorbances were read at 510 and 700 nm. The concentration of total monomeric anthocyanins was expressed as mg of cyanidin-3-*O*-glucoside equivalents per g of dry weight (mg CGE g^−1^ of DW) according to the following formula: TMA = (A × DF × MW)/(ɛ × C), where MW is the molecular weight of cyanidin-3-*O*-glucoside (449 g/mol); DF is the dilution factor; ε is the molar extinction coefficient of cyanidin-3-*O*-glucoside (29,600); C is the concentration of extracted volume; and A = (A_510_ − A_700_)pH_1.0_ − (A_510_ − A_700_)pH_4.5_.

### 4.4. Antioxidant Activity Assays

#### 4.4.1. ABTS^•+^ Radical-Scavenging Activity

The discoloration assay ABTS^•+^ (2,2’-azino-bis (3-ethylbenzothiazoline-6-sulphonic acid)) was used to determine the radical-scavenging activity of berries extracts according to Re et al. [56] and Stratil et al. [57]. To prepare the ABTS^•+^ work solution, in double distilled water, 7 mM ABTS and 140 mM K_2_S_2_O_8_ were used. The mixture was incubated for 12–16 h at room temperature in the dark; then, its absorbance was adjusted to 0.7–0.8 with absolute ethanol in a wavelength of 734 nm. Then, 15 µL of extract (or 70% methanol to measure the blank) plus 285 µL of the ABTS^•+^ work solution was mixed and put in the dark for 10 min, and finally, the absorbance was read at 734 nm. Using a Trolox calibration curve the results were expressed as µmol Trolox µg^−1^ of DW.

#### 4.4.2. DPPH Radical-Scavenging Activity

The radical-scavenging activity assay was carried out according to Brand-Williams et al. [58], Sánchez-Moreno et al. [59], and Siddhraju and Becker [60], combining 285 µL methanolic solution containing DPPH (2,2-diphenyl-1-picrylhydrazyl) radicals (10^−5^ mol/L) with 15 µL of extract. The mixture was vigorously shaken and left to stand in the dark for 30 min. The reduction of the DPPH radical was detected by measuring samples absorbance at 517 nm. The blank was made with 15 µL of 70% methanol and 285 µL of methanolic solution containing DPPH radicals. Using a Trolox calibration curve, the results were expressed as µmol Trolox µg^−1^ of DW.

#### 4.4.3. FRAP Assay

Our modification of the FRAP (ferric-reducing antioxidant power) assay was a modification of methods of Benzie and Strain [61] and Stratil et al. [57]. The FRAP reagent was prepared using 1 volume of an aqueous 10 mM solution of TPTZ (2,4,6-Tri(2-pyridyl)-s-triazine) in 40 mM HCl mixed with the 1 volume of 20 mM FeCl_3.6_H_2_O and 10 volumes of 300 mM acetate buffer, pH 3.6. Then, 25 µL of extract was mixed with 275 µL of FRAP reagent. The mixture was vigorously shaken and left to stand for 5 min in the dark, and the absorbance at 593 nm was recorded. The blank was made with 25 µL of 70% methanol and 275 µL of FRAP reagent. Using a Trolox calibration curve, the results were expressed as µmol Trolox µg^−1^ of DW.

### 4.5. Total RNA Extraction, cDNA Synthesis and Quantitative Real-Time PCR

To investigate the impact of the tested biostimulants on the secondary metabolism, the expression of several target genes (Table S5) encoding key enzymes involved in flavonoid biosynthesis and transport was analyzed. For this, we performed the extraction of total RNA from 100 mg of berry tissue using a Spectrum Plant Total RNA kit (Sigma-Aldrich, Darmstadt, Germany), following the manufacturer’s protocol. Quantity and quality of RNA were checked using a Nanodrop 2000 spectrophotometer (Thermo Scientific, Fremont, CA, USA) and 1% agarose gel electrophoresis stained with Midori Green Advance ^®^ (Nippon Genetics, Düren, Germany). Per each sample, total RNA reverse transcription was performed using 500 ng of RNA, SuperScript^®^ IV Reverse Transcriptase (Thermo Fisher Scientific, Waltham, MA USA), and a 1:1 mix of random primers and 50 µM oligo(dT)20 primers (Thermo Fisher Scientific, Waltham, MA USA), according to the manufacturer’s instructions. The differential gene expression of eight (*PAL*, *CHS*, *F3H*, *ANR*, *UFGT*, *ABCC1*, *MATE1*, and *GST*) genes involved in the secondary metabolism was carried out via real-time RT-PCR (Table S5, see Supplementary Material). Real-time RT-PCR was carried out on a Quant Studio 3 Real-Time PCR System (Thermo Fisher). Each reaction contained 500 nM of each primer, 4 µL of cDNA (1:10 dilution of the synthesis reaction), 10 µL of PowerUpTM SYBRTM Green Master Mix (Thermo Fisher, Waltham, MA USA), and water up to 20 µL. Each reaction was performed in triplicate. The applied thermal cycling conditions amounted to a hold stage at 50 °C for 2 min and 95 °C for 10 min, followed by 50 cycles: 95 °C for 20 s, 57 °C for 45 s, and 72 °C for 30 s. Finally, we introduced a melting curve stage at 95 °C for 15 s, 57 °C for 1 min, and 95 °C for 1 s to detect non-specific amplification in cDNA samples. Gene transcripts were quantified upon normalization to the reference gene *ubiquitin* (*UBI*) [62] by comparing the threshold cycle (Ct) of each target gene with *UBI* Ct. The relative quantification per each gene was calculated according to the 2^−ΔΔCt^ method, where ΔCt is the difference in threshold cycle between the average mean of the target and reference gene (*UBI*), and ΔΔCt is the difference between the average ΔCt of the target and control samples [63].

### 4.6. Statistical Analysis

Data were analyzed using SPSS Statistics for Windows (IBM SPSS Statistics for Windows, Version 23.0. Armonk, NY, USA: IBM Corp). The results were presented as the mean (n = 90 for quality assessment of fruits or *n* = 3 for the determination of phenolics, AA, and relative gene expression) with the standard error (SE). Statistical differences between treatments in each phenological stage were evaluated via one-way ANOVA, followed by Tukey’s multiple range test at (*p* < 0.05). One- and two-way ANOVA, establishing the phenological stage effects on the control and treated grapevines, were also performed. Principal component analysis (PCA) was carried out using ggbiplot (https://github.com/vqv/ggbiplot) R package. Clustered heatmap was carried out using gplots [64] R package. While, Pearson’s correlation between phenotypical and gene expression data was performed using psych R package [65].

## 5. Conclusions

Grapevine foliar sprayings are currently a mitigation strategy employed to alleviate climate change effects on grapevine development and on berries and wine quality. Although preliminary, the results obtained in cv. Touriga Franca, the major red variety cultivated in DDR, with the application of the biostimulants *A*. *nodosum* extract and glycine betaine, demonstrated improvements in several biochemical parameters, including increased contents of total phenols, flavonoids, *ortho*-diphenols, and anthocyanins. Alongside heightened expression levels of key genes involved in the secondary metabolism, ANE treatment exhibited a more pronounced influence during veraison, up-regulating *CHS*, *GST*, *F3H*, and *MATE1*, with a corresponding increase in flavonoids concentration. At harvest, the GB 0.1% treatment up-regulated *F3H* and *GST*, subsequently increasing the concentrations of total phenolics, flavonoids, *ortho*-diphenols, and anthocyanins. The foliar application of both biostimulants significantly improved the performance of *V. vinifera* by positively affecting physiological parameters and influencing secondary metabolism through elicitation, ultimately resulting in an improved grape quality. Further studies involving the additional growing seasons and time points of analysis and including formulations combining ANE and GB will provide valuable insights into the dynamics of the effects of these biostimulants on grapevine performance and quality.

## Data Availability

Data are contained within the article.

## Supplementary Materials

The following supporting information can be downloaded at: https://www.mdpi.com/article/10.3390/ijms25105335/s1.
