# Supplementary material for: Exploring Seaweed and Glycine Betaine Biostimulants for Enhanced Phenolic Content, Antioxidant Properties, and Gene Expression of Vitis vinifera cv. “Touriga Franca” Berries
Source: Int J Mol Sci. 2024 May 14;25(10):5335. doi: 10.3390/ijms25105335 (PMC11121377; doi:10.3390/ijms25105335)
Supplement: Supplementary file 1 [file ijms-25-05335-s001.zip › ijms-2976241-supplementary.pdf]

Table S1. Variation in the content of bioactive compounds: total phenolics, flavonoids, *ortho*-diphenols, and total anthocyanins, in berries of cv. 'Touriga Franca' under different treatments at veraison and harvest. Values are means ± SD. C – Control; ANE – seaweed extract; GB – glycine betaine.

Bioactive Compounds	Growth Stage/Year	C	ANE 0.05%	ANE 0.1%	GB 0.1%	GB 0.2%
Total Phenolics (mg GAE g^{-1} DW))	Veraison	27.04 ± 9.63	28.57 ± 5.59	27.53 ± 7.73	37.31 ± 3.52	39.82 ± 9.79
	Harvest	7.52 ± 1.55	15.04 ± 3.31	13.52 ± 3.29	17.41 ± 10.93	8.48 ± 3.02
Flavonoids (mg CE g^{-1} DW)	Veraison	13.04 ± 0.73	9.82 ± 1.50	18.88 ±7.16	13.48 ± 2.66	24.37 ± 3.20
	Harvest	4.78 ± 2.97	4.86 ± 0.92	5.69 ± 1.53	6.31 ± 1.53	4.65 ± 1.33
Ortho-diphenols (mg GAE g^{-1} DW)	Veraison	13.25 ± 5.52	14.24 ± 2.85	15.96 ± 2.27	18.77 ± 1.72	18.78 ± 4.45
	Harvest	5.43 ± 0.64	5.67 ± 1.03	6.06 ± 1.80	8.58 ± 1.56	5.88 ± 2.54
Total anthocyanins (mg GAE g^{-1} DW)	Veraison	1.94 ± 0.66	1.96 ± 0.44	1.76 ± 0.65	2.56 ± 0.85	1.11 ± 0.54
	Harvest	1.99 ± 0.51	3.10 ± 0.79	2.78 ± 0.51	4.16 ± 0.20	2.20 ± 0.55

Table S2. Antioxidant activity: $ABTS^{\bullet+}$ radical-scavenging activity, DPPH radical-scavenging activity and FRAP assay, in berries of cv. 'Touriga Franca', under different treatments at veraison and harvest. Values are means ± SD. C – Control; ANE – seaweed extract; GB – glycine betaine.

Antioxidant Activity	Growth Stage/Year	C	ANE 0.05%	ANE 0.1%	GB 0.1%	GB 0.2%
$ABTS^{\bullet+}$ (µmol Trolox/µg)	Veraison	35.41 ± 2.62	37.69 ± 1.87	33.04 ± 3.08	36.46 ± 2.90	35.80 ± 3.66
	Harvest	41.70 ± 5.08	41.61 ± 5.51	40.65 ± 6.22	39.37 ± 3.45	44.08 ± 5.79
DPPH (µmol Trolox/µg)	Veraison	54.71 ± 9.30	43.53 ± 3.12	51.29 ± 8.68	51.61 ± 8.81	57.52 ± 3.41
	Harvest	21.86 ± 3.05	24.07 ± 3.87	22.66 ± 8.22	27.39 ± 4.50	21.58 ± 6.80
FRAP (µmol Trolox/µg)	Veraison	60.26 ± 25.03	55.11 ± 11.27	76.40 ± 22.05	85.38 ± 32.66	105.41 ± 28.86
	Harvest	43.30 ± 6.53	43.67 ± 10.71	43.67 ± 12.84	56.40 ± 11.41	36.40 ± 10.03

Table S3. Relative gene expression in berries of cv. 'Touriga Franca', under different treatments at veraison and harvest. Values are means ± SD. C – Control; ANE– seaweed extract; GB – glycine betaine; T – Treatment; PS – Phenological Stage; *PAL – Phenylalanine ammonia-lyase*; *CHS - Chalcone synthase*; *F3H – Flavanone 3-hydroxylase*; *MATE1 - Tonoplast transporter*; *UFGT – UDP glucose: flavonoid 3-O-glucosyltransferase*; *ABCC1 – Anthocyanin transporter*; *ANR – Anthocyanidin reductase*; *GST – Glutathione S-transferase*

Gene	Growth Stage/Year	C	ANE 0.05%	ANE 0.1%	GB 0.1%	GB 0.2%
PAL	Veraison	1.00 ± 0.02	0.81 ± 0.30	0.29 ± 0.09	0.73 ± 0.39	1.29 ± 0.18
	Harvest	1.01 ± 0.20	0.04 ± 0.03	0.03 ± 0.01	0.01 ± 0.01	0.00 ± 0.00
CHS	Veraison	1.00 ± 0.10	9.48 ± 2.35	8.83 ± 2.49	18.79 ± 6.01	5.29 ± 0.32
	Harvest	1.03 ± 0.29	1.11 ± 0.22	0.18 ± 0.02	0.01 ± 0.01	0.12 ± 0.00
F3H	Veraison	1.00 ± 0.01	14.39 ± 7.08	5.87 ± 1.62	17.93 ± 5.93	1.75 ± 0.06
	Harvest	1.09 ± 0.57	0.26 ± 0.27	0.88 ± 0.76	0.76 ± 0.70	0.22 ± 0.01
MATE1	Veraison	1.12 ± 0.55	5.74 ± 0.78	0.58 ± 0.19	0.54 ± 0.63	0.40 ± 0.05
	Harvest	1.02 ± 0.27	0.58 ± 0.29	0.24 ± 0.11	1.07 ± 1.21	0.35 ± 0.04
UFGT	Veraison	1.01 ± 0.19	2.40 ± 0.61	2.98 ± 2.20	5.24 ± 1.47	1.58 ± 0.42
	Harvest	1.05 ± 0.37	0.30 ± 0.10	2.19 ± 1.52	0.05 ± 0.02	0.02 ± 0.02
ABCC1	Veraison	1.00 ± 0.02	1.17 ± 0.58	1.66 ± 0.48	1.21 ± 0.68	1.35 ± 0.37
	Harvest	1.01 ± 0.19	3.46 ± 1.37	0.91 ± 0.48	0.79 ± 0.37	0.35 ± 0.08
ANR	Veraison	1.10 ± 0.57	4.35 ± 2.08	0.90 ± 0.43	0.35 ± 0.20	1.80 ± 0.29
	Harvest	1.32 ± 0.13	0.11 ± 0.17	0.02 ± 0.01	0.00 ± 0.00	0.00 ± 0.00
GST	Veraison	1.12 ± 0.35	16.52 ± 20.03	44.52 ± 1.58	33.03 ± 8.02	7.70 ± 2.79
	Harvest	1.15 ± 0.72	1.40 ± 0.41	1.65 ± 1.58	6.73 ± 9.31	4.08 ± 0.42

Table S4. Applications of five foliar treatments, phenological stages, date and dosages. C – Control; ANE – seaweed extract; GB – glycine betaine.

Phenological Stage	Date	Treatment	Total of vines
Flowering (BBCH 65)	May 21th 2020	ANE 0.05% (2.5mL ANE + 5L water)	30
		ANE 0.1% (5mL ANE + 5L water)	30
		GB 0.1% (5g GB + 5L water)	30
		GB 0.2% (10g GB + 5L water)	30
		Control (5L water)	30
Pea size (BBCH 75)	June 4th 2020	ANE 0.05% (2.5mL ANE + 5L water)	30
		ANE 0.1% (5mL ANE + 5L water)	30
		GB 0.1% (5g GB + 5L water)	30
		GB 0.2% (10g GB + 5L water)	30
		Control (5L water)	30
Bunch closer (BBCH 77)	June 16th 2020	ANE 0.05% (2.5mL ANE + 5L water)	30
		ANE 0.1% (5mL ANE + 5L water)	30
		GB 0.1% (5g GB + 5L water)	30
		GB 0.2% (10g GB + 5L water)	30
		Control (5L water)	30
Veraison (BBCH 81)	July 10th 2020t	ANE 0.05% (2.5mL ANE + 5L water)	30
		ANE 0.1% (5mL ANE + 5L water)	30
		GB 0.1% (5g GB + 5L water)	30
		GB 0.2% (10g GB + 5L water)	30
		Control (5L water)	30

Table S5. Primer sequences , and annealing temperatures for the genes analyzed.

Gene		Primer sequence	Annealing temperature	Reference
PAL	*Phenylalanine ammonia-lyase*	F 5′ CCTACTGTTCAGAGCTCCAG 3′<br>R 5′ GCCACTAGGTATGTGGTAGACA 3′	57 °C	[22]
CHS	*Chalcone synthase*	F 5′ CACTCTTCGAACTCGTCTCT 3′<br>R 5′ CCACCAAGCTCTTCTCTATG 3′	57 °C	[22]
F3H	*Flavanone3-hydroxylase*	F 5′ CAGTGCAAGACTGGCGCGAGATCGTA 3′<br>R 5′ TAGCCTCAGACAACACCTCCAGCAACT 3′	57 °C	[22]
ANR	*Anthocyanidin reductase*	F 5′ CTGTCAGGTTCAGTCTCCAT 3′<br>R 5′ GTTGGGACTTTGTACTGAGG 3′	57 °C	[22]
UFGT	*UDP glucose: flavonoid 3-O-glucosyl transferase*	F 5′ TGCAGGGCCTAACTCACTCT 3′<br>R 5′ GCAGTCGCCTTAGGTAGCAC 3′	57 °C	[22]
ABCC1	*Anthocyanin transporter*	F 5′ CTCCACTGGTCCTCTGCTTC 3′<br>R 5′ AGCCTGCTTCGAAAGTACCA 3′	57 °C	[22]
MATE1	*Tonoplast transporter*	F 5′ TGCTTTTGTGATTTTGTTAGAGG 3′<br>R 5′ CCCTTCCCCGATTGAGAGTA 3′	57 °C	[22]
GST	*GlutathioneS-transferase*	F 5′ AAGGATCCATGGTGATGAAGGTGTATGGC 3′<br>R 5′ AACTGCAGAAGCCAACCAACCAACAAAC 3′	57 °C	[22]
UBI	*Ubiquitin*	F 5' TCTGAGGCTTCGTGGTGGTA 3′<br>R 5' AGGCGTGCATAACATTTGCG 3′	57 °C	[56]

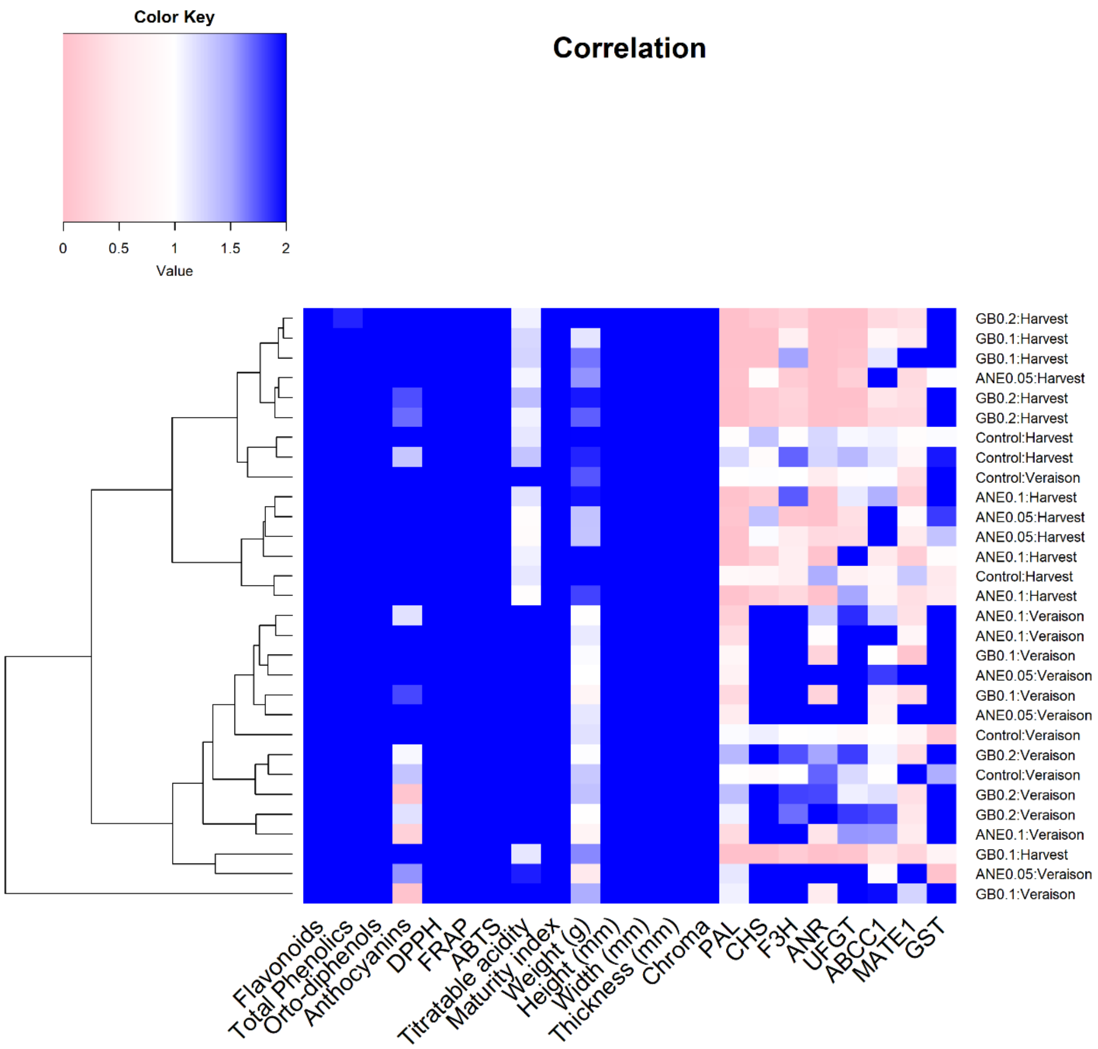

Figure S1. Heatmap of correlations between analyzed parameters at veraison and harvest in the tested different treatments. ANE– seaweed extract; GB – glycine betaine.

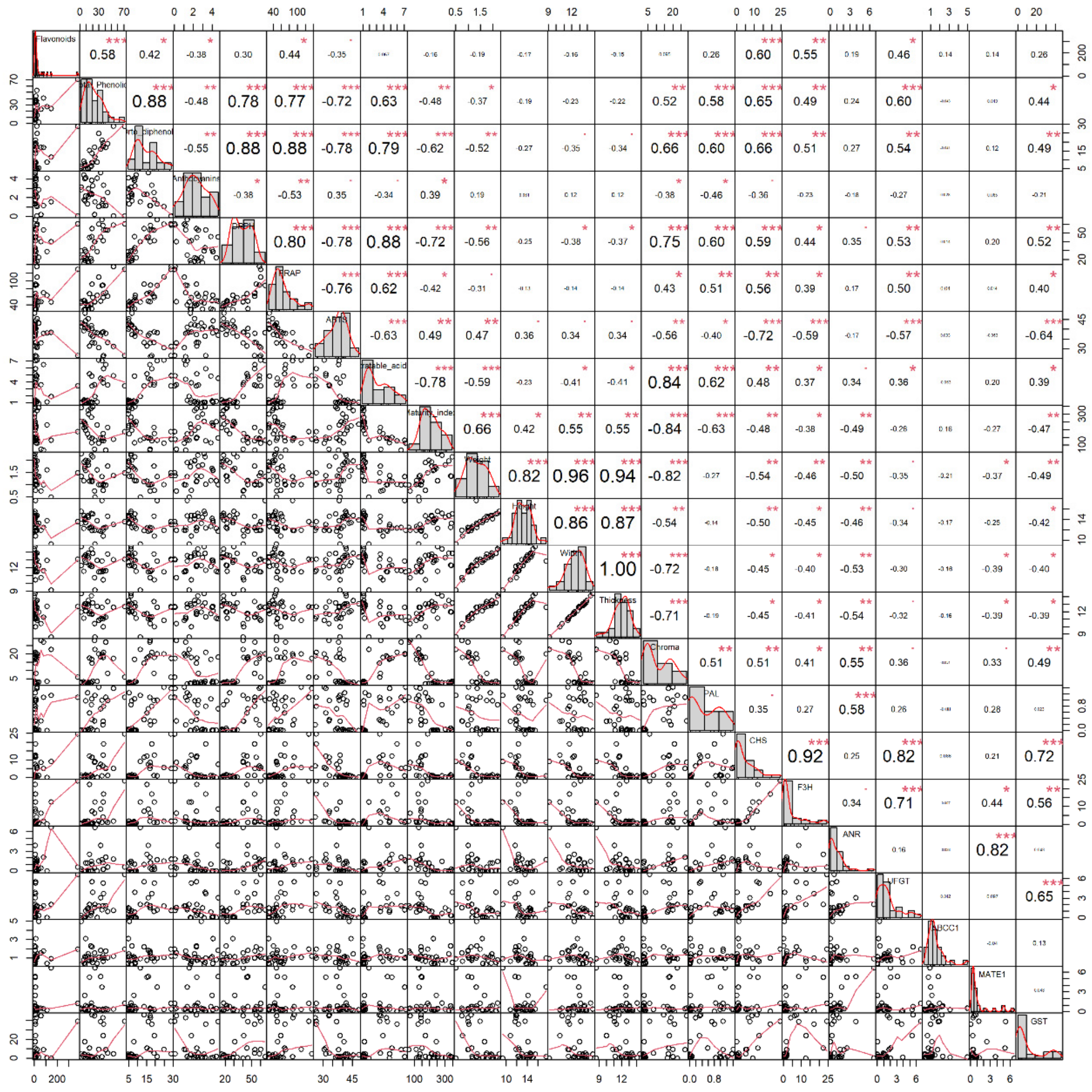

Figure S2. General correlations of the berry-related traits analyzed.
